# Perceptual punctuation: fixational eye movements reveal segmentation of auditory streams

**DOI:** 10.3389/fnins.2026.1731980

**Published:** 2026-01-23

**Authors:** Vincenzo Rizzuto, Oren Kadosh, Roberto Montanari, Yoram Bonneh

**Affiliations:** 1 International School of Advanced Science, University of Camerino, Camerino, Italy; 2 Department of Surgical, Medical and Molecular Pathology and Critical Care Medicine, Pisa, Italy; 3 Department of Ophthalmology, Riga Stradins University, Riga, Latvia; 4 School of Optometry and Vision Science, Bar-Ilan University, Ramat-Gan, Israel; 5 Institute of Pharmacology, Heidelberg University Hospital, Heidelberg, Germany; 6 The Leslie and Susan Gonda Multidisciplinary Brain Research Center, Bar-Ilan University, Ramat Gan, Israel

**Keywords:** active sensing, auditory scene analysis (ASA), auditory stream, fixational eye movements, microsaccade, oculomotor inhibition, perceptual bistability, temporal attention

## Abstract

**Introduction:**

Perception operates as rhythmically structured sampling in which temporal predictions determine when incoming signals are weighted. Fixational eye movements carry opposing consequences, enhancing acuity yet inducing brief peri-saccadic suppression, suggesting that their timing is paced by expected, salient rhythms. Auditory scenes can be parsed into competing streams that unfold over time. If fixation dynamics are shaped by temporal expectation, and auditory streaming imposes a percept-dependent temporal structure on otherwise identical acoustics, then fixational eye movements might provide a window into how listeners parse sound over time. We asked whether fixational eye movements reflect the perceived rather than the physical temporal organization of an ambiguous ABA– pattern.

**Methods:**

While listeners fixated and either attended High, Low, or All tones (Experiment 1, *n* = 15) or freely reported their percept (Experiment 2, *n* = 15), we recorded binocular eye position (500 Hz) and quantified microsaccade (MS) dynamics and eye-velocity spectra.

**Results:**

Across both experiments, eye-velocity spectra showed a percept-dependent redistribution between 2 and 4 Hz, with relative power shifting with the instructed/reported stream. A normalized 4–2 Hz index (ΔPSD) separated Low-tone from High-tone percepts across procedures. Time-resolved analyses further revealed within-trial waxing-and-waning of 2 vs. 4 Hz dominance, consistent with bistable fluctuations in maintaining a stream. Moreover, microsaccade reaction time (msRT), aligned to the onset of the sound sequence, differed significantly depending on the percept.

**Discussion:**

These findings extend oculomotor inhibition beyond discrete events, positioning fixation dynamics as a sensitive, report-free marker of auditory scene organization. We discuss mechanistic links to temporal attention and active sensing, and implications for a multisensory timing framework.

## Introduction

Living organisms survive by detecting change, predicting when it will happen, and acting at the right moment. A prey that hears the first crack of a twig or a hunter listening for the next footfall must bind what is occurring in the scene to when it occurs. That spatio-temporal binding is supported by an incessant stream of tiny eye movements, microsaccades, ocular drifts, and blinks, that punctuate perception even while gaze is nominally fixed (Rolfs, 2009; Martinez-Conde et al., 2004; Rucci and Victor, 2015). These minute displacements are embedded in a continuous perception–action loop that keeps sensory sampling in register with an ever-changing world (Ahissar and Assa, 2016; Rucci and Victor, 2015; Bonneh et al., 2015; Gruber and Ahissar, 2020; Ahissar et al., 2016). Microsaccades (MS) are rapid (< 1°), small-amplitude saccades that can be executed voluntarily, but during ordinary fixation they arise involuntarily at a rate of ~1–3 s^−1^ and display a characteristic inhibition–rebound pattern after every transient stimulus (Engbert and Kliegl, 2003; Rolfs, 2009). The latency of the first MS released from this inhibition, the microsaccade response time (msRT), is influenced by the stimulus parameters (e.g., contrast), attention, and expectation (Rolfs, 2009; Bonneh et al., 2013; Yuval-Greenberg et al., 2014; Bonneh et al., 2015; Kliegl et al., 2009), making it a sensitive oculomotor index of salience (Bonneh et al., 2015). Because msRT reflects how quickly oculomotor activity recovers after suppression, it serves as a sensitive index of perceptual salience and cognitive engagement (Bonneh et al., 2015; Denison et al., 2019; White and Rolfs, 2016).

### Benefits and costs of microsaccades

Microsaccades (MS) are not random eye twitches but precisely timed sampling movements that sustain perception. They enhance visual performance by preventing perceptual fading through continual retinal refresh (Ditchburn and Ginsborg, 1952; Martinez-Conde et al., 2006; Troncoso et al., 2008), by driving robust reafferent responses that modulate neuronal firing in the lateral geniculate nucleus and primary visual cortex (V1) (Martinez-Conde et al., 2002; Troncoso et al., 2015; Nativ et al., 2025), and by displacing the image by only a few arcminutes to scan the foveola maximizing spatial resolution (Ko et al., 2010; Poletti et al., 2013; Intoy and Rucci, 2020; Rolfs, 2009; Chen and Hafed, 2013, 2017).

Yet each MS also inserts a brief sensory pause, a fleeting moment of suppression in which visual sensitivity drops (Hass and Horwitz, 2011; Hafed and Krauzlis, 2010; Loughnane et al., 2018; Beeler, 1967).

This benefit–cost trade-off implies that MS should be strategically scheduled so that suppression does not coincide with behaviorally critical moments. Accordingly, microsaccade rates are transiently inhibited, the so-called *oculomotor inhibition*, before expected events in synchrony with attention and prediction (Denison et al., 2019; Amit et al., 2019; Kadosh and Bonneh, 2022a) consistent with a scheduling strategy that minimizes peri-microsaccadic sensitivity costs (Denison et al., 2019; Amit et al., 2019; Abeles et al., 2020; Badde et al., 2020).

Behaviorally, both humans and macaques suppress MS before expected targets; blinks and larger saccades show similar anticipatory inhibition. When this suppression fails, i.e., when a blink or a (micro)saccade occurs around the expected target, it signals lapses or altered temporal attention and predicts poorer performance, and in clinical populations it tracks state and medication effects (Denison et al., 2019; Betta and Turatto, 2006; Fried et al., 2014; Beker et al., 2025; Dankner et al., 2017).

The depth and timing of this inhibition scale with temporal attention/expectation, stimulus salience, familiarity, and deviance/surprise (Denison et al., 2019; Amit et al., 2019; Rosenzweig and Bonneh, 2019; Kadosh and Bonneh, 2022a, 2022b).

These sensorimotor trade-offs are supported by modulations along the visual pathway (Hass and Horwitz, 2011; Herrington et al., 2009; Hafed et al., 2009; Krauzlis et al., 2017; Chen et al., 2015), although the present study focuses on behavioral dynamics rather than neural mechanisms.

In other words, every MS inserts a fleeting blind interval into the visual stream. How does the brain schedule these unavoidable gaps so that critical information is not lost?

As Miles Davis observed, “In music, silence is more important than sound” (Davis and Troupe, 1989); by analogy, perception may be orchestrated as a score in which sensory “rests” correspond to brief peri-microsaccadic intervals of reduced visual sensitivity that help structure an otherwise continuous stream of sampling. Microsaccades are the events that mark these pauses and, in this sense, may act like commas, rhythmically segmenting perceptual flow.

### Beyond vision: auditory oculomotor coupling

Crucially, microsaccade (MS) timing is not confined to the visual domain. In auditory oddball paradigms, rare or deviant tones elicit a prolonged microsaccadic inhibition compared to standards, reflecting rapid detection of novelty (Valsecchi and Turatto, 2009; Widmann et al., 2014; Kadosh and Bonneh, 2022b). MS dynamics can even differentiate auditory targets from non-targets within ~150 ms, indicating that oculomotor behavior participates in early stages of auditory categorization (Widmann et al., 2014). Moreover, the depth and latency of inhibition scale with deviance magnitude: larger pitch differences trigger earlier inhibition onset and longer rebound delays, an effect that depends on the inter-trial interval and reflects both auditory salience and deviance processing (Kadosh and Bonneh, 2022b).

Beyond brief events, MS rate also indexes sustained auditory states (Leszczynski et al., 2023). It decreases during episodes of musical absorption (Lange et al., 2017), correlates with the moment-to-moment attentional effort required for speech-in-noise comprehension (Contadini-Wright et al., 2023) and is sensitive to abstract linguistic structure implicating higher-order lexical evaluation in the auditory modality (Kadosh et al., 2025). Related studies have shown that cross-modal attention modulates oculomotor inhibition even in the absence of visual input, underscoring tight coupling between auditory attention and gaze stabilization (Zhao et al., 2024; Denison et al., 2019). However, most of this literature links oculomotor inhibition to externally defined, discrete auditory events (e.g., deviants or targets) and therefore does not address whether fixational eye movements track temporal structure that is internally determined by perceptual organization when the acoustic input is unchanged. Thus, beyond demonstrating cross-modal coupling, an open question is whether fixation dynamics can be leveraged as a report-free readout of how the auditory system parses an ambiguous stream over time. More broadly, such dynamics may reflect a cross-modal active-sensing loop.

Together, these findings support the idea that fixational eye movements carry information about the saliency of individual sounds and, potentially, about the temporal organization of complex acoustic scenes. This raises the possibility that fixation dynamics provide a continuous, cross-modal marker of percept-dependent temporal grouping.

According to the framework of Auditory Scene Analysis (Bregman, 1990), the auditory system decomposes complex acoustic mixtures into perceptual streams that evolve over time. If microsaccade timing is governed by temporal expectation, and auditory scenes possess a temporal structure that is at least partly perceived rather than purely physical, then fixational eye movements might provide a unique window into this internal, percept-dependent structuring of sound. A canonical paradigm employs the alternating ABA– pattern, in which two tones (A and B) are presented in a repeating triplet at a constant tempo. Depending on frequency separation, presentation rate, and attention, listeners perceive either a single “galloping” stream or two segregated streams, a form of auditory bistability in which perception alternates spontaneously while the physical stimulus remains fixed (Barniv and Nelken, 2015; Higgins et al., 2020, 2023).

Auditory bistability in the ABA– paradigm typically unfolds in two phases: an initial build-up/commitment period over the first seconds followed by stochastic alternations whose dominance durations span seconds to tens of seconds with substantial between-listener variability. Transitional intervals can include weak dominance or report uncertainty, motivating the treatment of unstable/mixed epochs separately from stable perceptual states.

Here we leverage this bistability to test whether fixation dynamics track the perceived temporal organization of the ABA– sequence under identical acoustics, moving beyond event-locked oculomotor inhibition to a continuous readout of perceptual grouping over time.

Like the Necker cube in vision, auditory bistability reveals how perceptual organization can arise from internal dynamics rather than stimulus change. Notably, the temporal dynamics of alternations in auditory and visual bistability show common principles of perceptual organization (Pressnitzer and Hupé, 2006), motivating the idea that a cross-modal, report-free marker might track these internally driven fluctuations over time.

In the visual domain, MS have been shown to influence and sometimes trigger perceptual reversals in ambiguous motion and binocular rivalry (van Dam and van Ee, 2005; Laubrock et al., 2008; Troncoso et al., 2008; Bonneh et al., 2010), indicating that oculomotor behavior can play an active role in resolving perceptual ambiguity. Although the onset of oculomotor inhibition is tightly time-locked to external transients, its release, the timing of the subsequent MS, is modulated by higher-order factors such as task difficulty, temporal predictability, working-memory load, and stimulus familiarity (Bonneh et al., 2013, 2015; Siegenthaler et al., 2014; Amit et al., 2019; Rosenzweig and Bonneh, 2019; Shwartz and Bonneh, 2023).

Whether an analogous mechanism operates in audition, such that fixational eye-movement dynamics track the perceived (stream-dependent) temporal structure of sound under unchanged acoustics, rather than merely responding to discrete external transients, remains unknown.

### Present study

In the present study, we tested whether fixational eye movements entrain to the perceived, rather than the physical, rhythm of a bistable ABA– auditory sequence. We analyzed both the timing of MS and the spectral structure of eye-movement velocity during steady fixation to determine whether oculomotor activity reflects perceptual organization in the absence of overt reports. Specifically, we predicted that MS probability, MS reaction time (msRT; the latency from triplet onset to the first MS), and spectral power would align with the rhythm of the dominant percept: 2 Hz when the high-frequency stream dominates, and 4 Hz when the low-frequency stream dominates. For the integrated All (“galloping”) percept the perceptual meter is not uniquely constrained *a priori*: in principle it could emphasize the overall triplet cycle (2 Hz) and/or a within-cycle subdivision (e.g., 4 Hz). Accordingly, we treat the relevant temporal structure in the All percept as a testable question and assess it empirically by quantifying how eye-velocity spectra redistribute power between 2 and 4 Hz across instructed/reported percepts.

To test this, Experiment 1 employed selective-attention instructions (“attend high,” “attend low,” or “attend all”) to bias perception while maintaining fixation. Because sustaining a single percept for extended (20 s) sequences is difficult, Experiment 2 replicated the paradigm with shorter (5 s) trials in which participants reported their dominant percept after each sequence. Together, these experiments test whether involuntary eye movements encode perceptual rhythm across both instructed and self-reported conditions, establishing fixational eye behavior as a report-free index of auditory perceptual segmentation and extending active sensing principles from vision to audition.

## Methods

### Participants

A total of seventeen participants took part in Experiment 1 (8 female, 9 male; aged 20–40) and eighteen in Experiment 2 (9 female, 9 male). All were students at Bar-Ilan University and naive, except for one of the authors. Two participants were excluded from Experiment 1 due to poor recording quality or because their dataset contained fewer runs than planned. Three participants were excluded from Experiment 2 because they contributed an insufficient number of trials (fewer than five) in at least one condition, since not all participants reported experiencing every percept.

All participants had normal or corrected-to-normal vision, reported normal hearing (self-report), none reported a history of hearing impairment and were naïve to the purpose of the study. Both experiments were approved by the Bar-Ilan University Ethics Committee and written informed consent was obtained from all participants. Furthermore, all methods and procedures were conducted in compliance with the relevant guidelines and regulations.

### Apparatus

Auditory stimuli were presented through headphones at 70 dB SPL. Participants sat 0.6 m from a 24-inch, 100 Hz LCD monitor in a dimly lit room, with their heads stabilized by a combined chin-and-forehead rest. The experiment ran on an in-house psychophysics platform (PSY; Y. S. Bonneh). Eye movements were recorded binocularly with a remote camera–based system (EyeLink 1,000, SR Research) running at 500-Hz sampling rate, equipped with a 35 mm lens positioned 0.52 m from the participant. Each session began with a standard 9-point calibration. All recordings were conducted binocularly, but the analyses were performed monocularly using data from the left eye. This approach was chosen based on our experience with the accuracy of microsaccade detection, as reported in our previous studies (Yablonski et al., 2017; Bonneh et al., 2015; Bonneh et al., 2016).

### Stimuli

**Figure 1:**
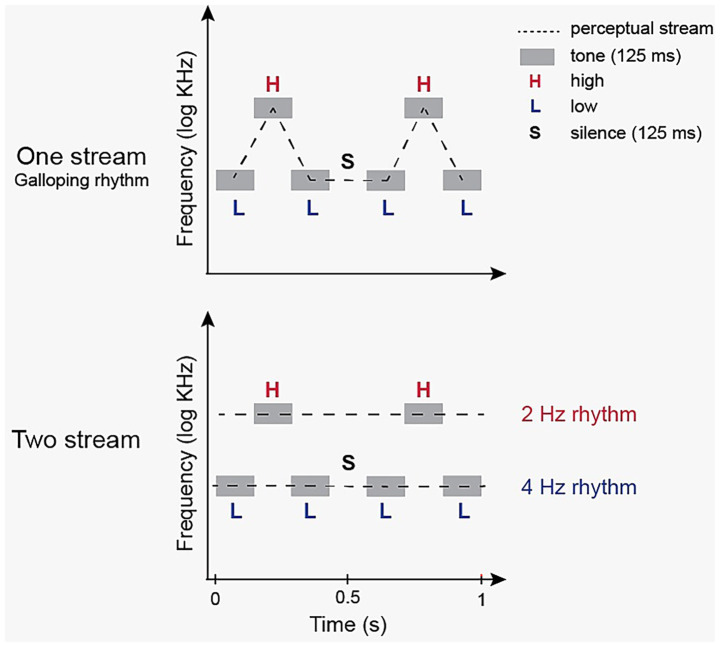
Stimulus illustration: perceptual auditory streams. Schematic time–frequency plots illustrate how Δf and rate govern organization. Left: With small Δf/slow rate, nearest-neighbor relations link alternating frequencies, favoring a single “galloping” stream. Right: With large Δf/faster rate, tones group by frequency, yielding two segregated streams (high vs. low). Our stimulus (L = 500 Hz, H = 1,400 Hz) presented as ABA– at 2 Hz (375 ms triplet + 125 ms gap) produces a bi-stable regime in which perception flips between integrated (2 Hz) and segregated (4 Hz) organizations; within each triplet, the H tone recurs once (2 Hz) whereas the L tone recurs twice (4 Hz).

Auditory streaming was elicited with the classic A–B–A pattern from Bregman’s auditory scene analysis. In this paradigm, two pure tones of different frequency (A and B) are arranged in repeating triplets A–B–A at a constant tempo. Perceptual organization depends jointly on the frequency separation (Δf) and the presentation rate: small Δf and/or slow rate favor a single, integrated “galloping” stream; large Δf and/or faster rate favor two segregated streams (high vs. low), and intermediate combinations yield bi-stable perception with spontaneous alternations between the two organizations (Bregman, 1990) (see Figure 1).

In our implementation, we used A = 500 Hz and B = 1,400 Hz (Δf ≈ 900 Hz, ~1.5 octaves) and presented the sequence at a 2 Hz cycle (one triplet every 500 ms). Each ABA– triplet lasted 375 ms and was followed by a 125 ms silent gap, yielding the 2 Hz rhythm. Critically, within each triplet, the high-frequency B tone occurs once (effectively a 2 Hz stream), whereas the low-frequency A tone occurs twice (effectively a 4 Hz stream). This asymmetry is what allows selective attention, or the dominant spontaneous percept, to reveal itself as entrainment at 2 Hz (High-tone stream) or 4 Hz (Low/All streams).

Stimuli were presented binaurally over headphones at 70 dB SPL in a dim, quiet room. Participants sat 60 cm from a 24″, 100 Hz LCD monitor used for fixation cues.

## Procedure

Participants maintained central fixation while listening to the continuous ABA– sequence. Eye position was recorded binocularly with head stabilized by a combined chin-and-forehead rest; a 9-point calibration preceded each session.

We ran two experiments with the same acoustic pattern but different task demands:

- Experiment 1 (20-s blocks). Participants completed six runs, each consisting of one 20-s block. There were two runs per condition (High, Low, All), for a total of six blocks per participant. Within each 20-s block, forty ABA– triplets were presented. On each run, participants were instructed to fixate and attend to either the High, Low, or All tones (ABA- galloping percept) and to maintain the requested percept for as long as possible. The order of conditions across runs was randomized for each participant.
- Experiment 2 (5- s blocks). Participants completed three runs, except for two participants which ran the experiment twice. Each run was constructed of 32 short blocks. Within each block, ten ABA– triplets were presented. After each 5-s block, participants reported their dominant percept (High, Low, All, or Confused, indicating an unstable or fluctuating percept).

### Data analysis

#### Microsaccade detection and preprocessing

MS were identified with the velocity-based algorithm of Engbert and Kliegl (2003), the same procedure used in our previous studies (Yablonski et al., 2017; Bonneh et al., 2015; Kadosh and Bonneh, 2022a; Kadosh and Bonneh, 2022b; Kadosh and Bonneh, 2023). Prior to detection, raw gaze traces were smoothed with a 15 ms LOWESS filter, which improves microsaccade yield in noisier recordings (Engbert and Kliegl, 2003). We included only eye movements with a peak velocity range of 8–250^o^/s, an amplitude between 0.08 and 1^o^, and a duration that exceeded 9 ms. Eye blinks were flagged as in our previous study (Bonneh et al., 2016): intervals of zero pupil size were marked and then extended to cover the eyelid-closure and opening periods, estimated from the vertical eye movement that precedes the blink (Yablonski et al., 2017).

For Experiment 1, each 20-s eye-tracking block was epoched from −0.1 to 3 s relative to every triplet onset, yielding forty overlapping epochs per block. Although each sound pattern lasted 375 ms followed by a 125 ms silent gap, the longer epoch accommodates the sliding-window spectral analysis applied later. For Experiment 2, we used a single epoch per block, starting 0.9 to 5 s relative to the block onset, to avoid noisy beginnings, as perception builds gradually. Short segments of missing data within an epoch (e.g., during blinks) were linearly interpolated before the spectral computations.

#### Calculation of the microsaccade rate function and reaction time

The MS rate modulation function was calculated as in our previous studies (Yablonski et al., 2017; Bonneh et al., 2010; Rosenzweig and Bonneh, 2019). The rate was calculated based on the raw onset times relative to each triplet-stimulus, within a window spanning from 100 ms before to 3 s afterwards. MS onsets in this window were converted into a continuous rate signal for each epoch by convolving a 500/s eye-tracker sampling rate with a Gaussian kernel (*σ* = 50 ms) at the time of each onset, assuming an estimate of only one microsaccade per sample duration for a sampling rate of 500 Hz. Traces were first averaged across trials within each participant, then each participant’s overall mean was subtracted from their trace. These adjusted traces were averaged across participants and finally shifted by adding back the grand mean (computed across participants and conditions) to retain an interpretable scale. Subtracting each participant’s mean reduces the influence of participants with unusually large overall amplitudes, making it less likely that the observed effect is driven by a small number of outliers. Importantly, these rate plots are intended only to visualize the temporal dynamics of MS inhibition and rebound. The long, partially overlapping epochs accentuate the rhythmic structure of MS behavior. For statistical analysis we measured the microsaccade reaction time (msRT), as the latency of the first MS occurring between 0 and 500 ms after stimulus onset.

#### Spectral analysis

To quantify rhythmic structure in the eye-movement velocity trace (which predominantly reflects MS but also includes slow drift) we performed a Lomb–Scargle periodogram using MATLAB’s plomb function. For each trial we analyzed the full epoch 0–3 s or 0–5 s post-stimulus segment, depending on the experiment, and extracted power at the two task-relevant frequencies, 2 Hz (the perceived rhythm in the High condition) and 4 Hz (the perceived rhythm in the Low condition).

Power was summarized within a ± 0.5 Hz window centered on each frequency (1.5–2.5 Hz and 3.5–4.5 Hz, respectively). Within each window we computed a normalized “peak-to-background” metric:

$$P(f_{0})=\frac{\max[P(f_{0}\pm 0.5\;\mathrm{Hz})]-\text{mean}[P(f_{0}\pm 0.5\;\mathrm{Hz})]}{\text{mean}\;[P(f_{0}\pm 0.5\;\mathrm{Hz})]}$$

where *f_0_* is either 2 Hz or 4 Hz and *P(f)* denotes spectral power at frequency *f*. This measure captures how prominently the peak at *f_0_* rises above its local baseline while scaling out overall power differences across participants. Identical calculations were applied for both frequencies, yielding a pair of normalized power values per trial that were carried forward to group-level statistics.

#### Area under the curve analysis

To demonstrate how participants struggled to sustain the instructed percept over the 20-s sequence of Experiment 1, we tracked temporal fluctuations in the 2- and 4-Hz spectral components. The eye-movement velocity trace was parsed into partially overlapping epochs, each assigned to the chronological time corresponding to its midpoint. For every epoch we extracted the Lomb–Scargle power at 2 Hz and 4 Hz (see “Spectral analysis” above), yielding a time-resolved power series for each participant and perceptual condition. We then computed, for each series, the mean trajectory and its area under the curve (AUC) as summary measures of sustained entrainment. Finally, individual time courses were averaged to produce group-level curves that visualise how rhythmic power waxes and wanes across the 20-s interval.

#### Statistical assessment

**Figure 2:**
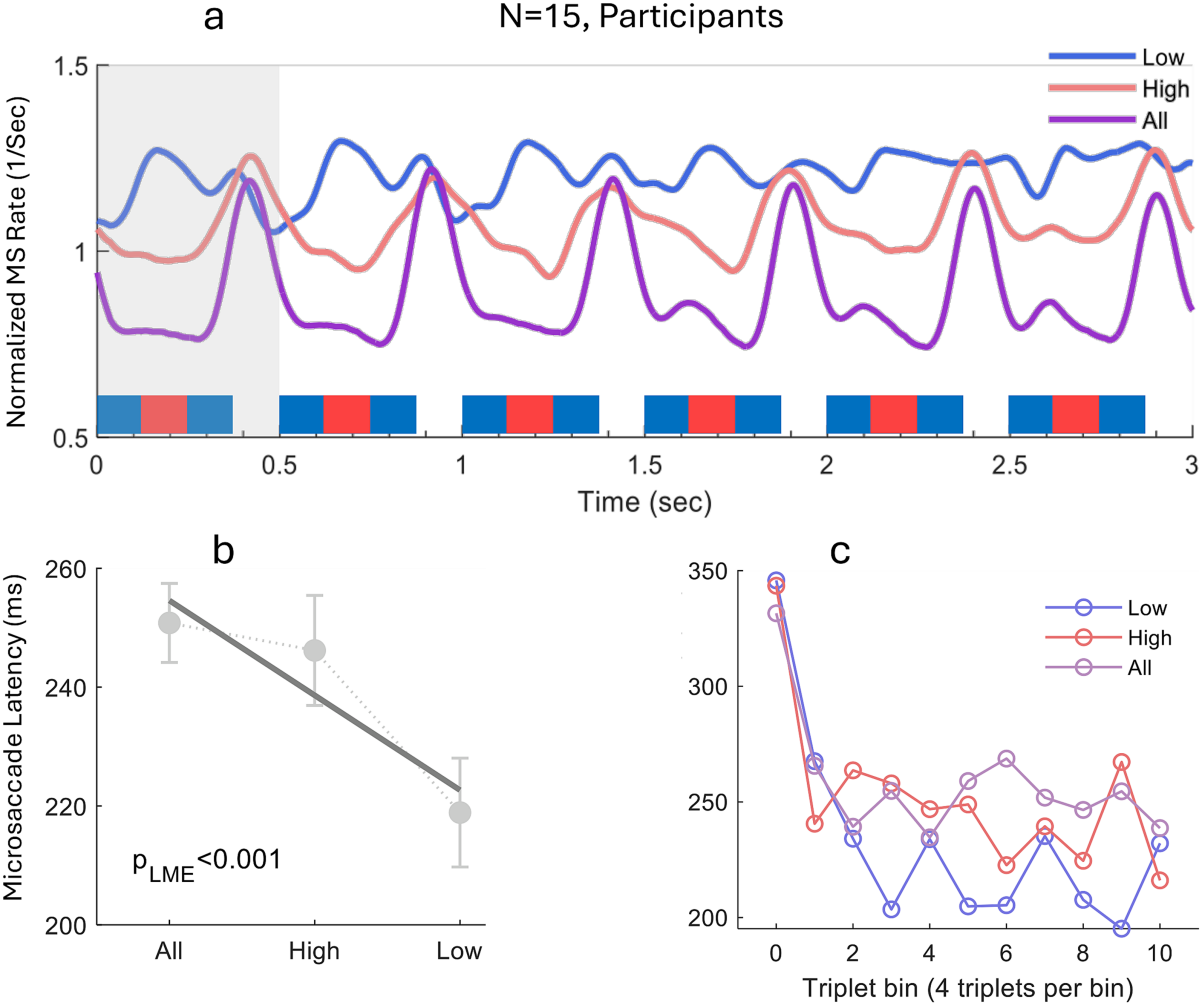
Experiment 1: microsaccade dynamics across auditory-stream conditions. The figure provides a demonstration of microsaccade behavior over time across the three experimental conditions: low-tone, high-tone and both-tones (All) perceptual states. **(a)** Normalized microsaccade rate modulation averaged across observers over 3 s partially overlapping epochs triggered by sound-pattern-onset (blue rectangles, see the methods for the normalization method). It illustrates the pattern of microsaccade inhibition and release with a faster microsaccade latency and a second rebound for the low-tone condition. The grey band marks the 0–500 ms window used for latency estimation. **(b)** Latency of the first MS released from inhibition (msRT) after each sound pattern, which triggers an inhibition with different duration depending on perceptual content. Statistical evaluation performed by LME (see the Methods) shows a significant effect of perceptual streams, *p* < 0.001, LME. **(c)** Latency of the first MS following each triplet, computed across the block and averaged in bins of four consecutive triplets. A linear trend over bins was significant in the Low condition (*p* < 0.05), marginal in the High condition (*p* < 0.1), and not significant in the all condition (*p* > 0.1), using LME.

For the MS-rate time courses in Experiment 1 (Figure 2A), we tested whether the apparent condition differences exceeded chance using a Monte-Carlo permutation procedure. For each participant, MS rate (events/s) was computed in 20-ms bins from −0.1 to 3 s relative to sound-pattern onset and averaged across trials of each instruction condition (Low, High, All). To obtain a null distribution, condition labels were randomly permuted within participants 1,000 times, the group-mean condition differences in MS rate were recomputed for each time bin, and the maximum absolute difference across time was stored on each iteration. The observed time course was then compared to this max-statistic null distribution to derive a two-tailed, family-wise–corrected *p*-value for each bin.

Microsaccade reaction time (msRT) was evaluated with a linear mixed-effects model (LME) in which perceptual condition served as a fixed effect and participant as a random intercept. Normalized spectral-power, by demeaning within participants (subtracting the participants overall mean), and adding the global mean of all participants and conditions, were submitted to a two-way repeated-measures ANOVA. The within-subject factors Frequency (2 Hz vs. 4 Hz) and Perceptual Condition (Low vs. High vs. All) were used followed by a multiple comparison test with Tuckey-Kramer. To quantify frequency-specific bias directly, the difference score ΔPower = Power₄ − Power₂ was tested against zero with a paired-samples t-test. All statistical tests were two-tailed with *α* = 0.05; effect sizes for *t*-tests we used Cohen’s d.

## Results

### Experiment 1

We first examined the microsaccade (MS) rate time course in each condition to visualize the inhibition–rebound dynamics evoked by the ABA– pattern. Eye-tracking epochs were extracted around every sound-pattern onset, spanning from 100 ms before the stimulus to 3 s afterwards, resulting in 40 epochs per block. Because successive epochs overlap, the resulting MS-rate time course is amplified (see Methods) and is shown in Figure 2A for descriptive purposes. Each sound pattern elicited the typical microsaccade inhibition and rebound sequence, yet the profile visually differed between conditions. In the Low-tone condition, inhibition was shorter and followed by a second, smaller rebound near pattern offset. The High-tone condition exhibited a deeper, longer inhibition with a single rebound at stimulus offset. The full pattern, All-tones condition combined features of the other two, showing a prolonged inhibition interrupted by a modest mid-pattern rebound. Although MS probability showed condition-specific fluctuations, a Monte-Carlo permutation test comparing the observed modulations revealed no significant effect between either percept; these plots are therefore presented for illustration of MS timing only (see the Methods).

To quantify these differences, we measured the latency of the first MS released from inhibition (msRT) within 0–500 ms after stimulus onset and analyzed these msRTs with a linear mixed-effects model. As shown in Figure 2B, the LME revealed a significant effect of perceptual condition on msRT (*p* < 0.001); descriptively, msRTs were shortest in the Low-tone condition, consistent with faster recovery of oculomotor activity when attention was directed to the faster, low-pitch stream.

To examine whether the msRT changed over the course of a block, we averaged values in consecutive bins of four triplets (Figure 2c). A linear mixed-effects model testing latency as a function of bin index revealed a significant within-block trend in the Low-tone condition (*p* < 0.05), a marginal trend in the High-tone condition (*p* < 0.1), and no reliable trend in the All condition (*p* > 0.1).

**Figure 3:**
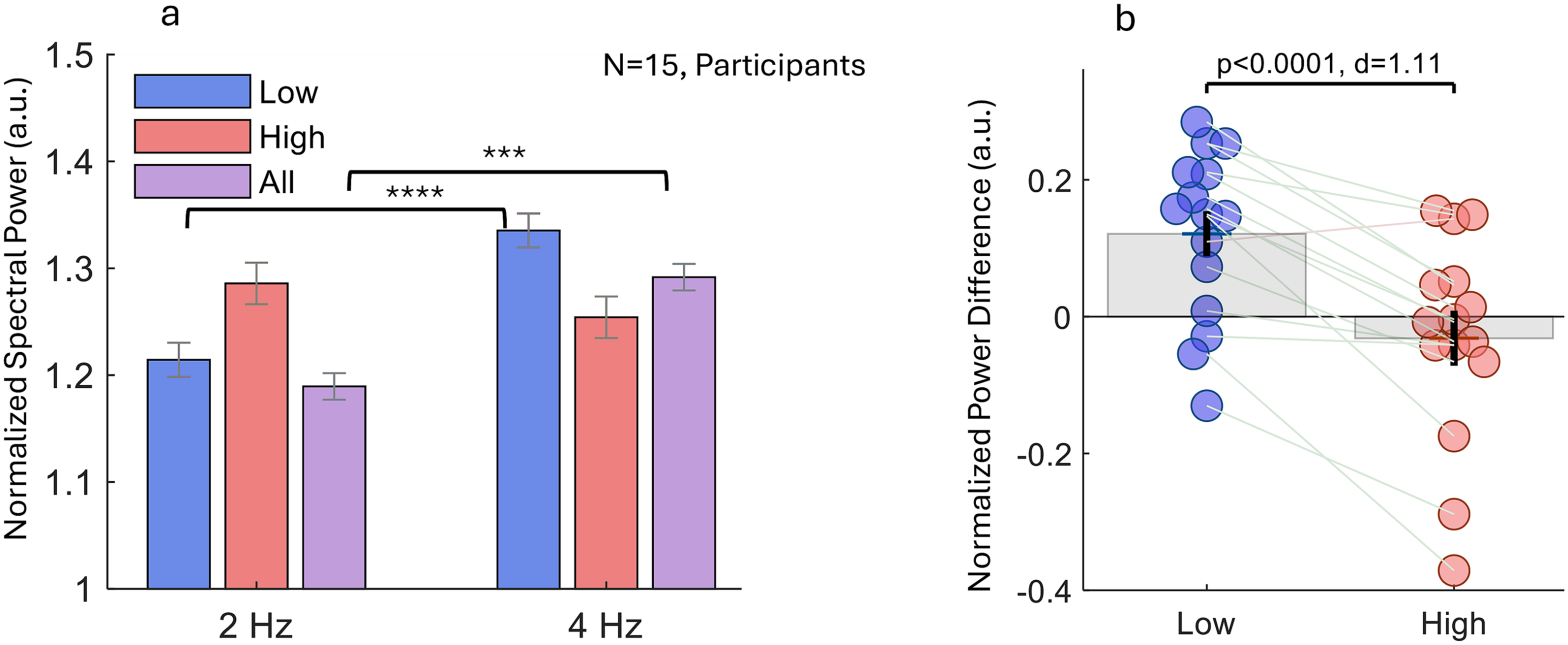
Experiment 1: spectral characteristics of eye-velocity modulation. **(a)** Group-mean power-spectral density (PSD), normalized within participants (see the Methods), reveals that oculomotor activity shifts toward the 2 Hz component when attention is on the *High-tone* stream and toward the 4 Hz component when attention is on the *Low-tone* stream or on the full triplet *All-tones*. A two-way repeated-measures ANOVA (Frequency: 2 Hz vs. 4 Hz; Condition: Low, High, All) showed a significant main effect of frequency (*F*(1,84) = 23.3, *p* < 0.00001) and a significant Frequency × Condition interaction (*F*(2,84) = 13.2, *p* < 0.00001). Asterisks mark post-hoc contrasts: *p* < 0.001 (***) and *p* < 0.0001 (****). **(b)** Individual ΔPSD values (4 Hz – 2 Hz) for the Low-tone and High-tone conditions. Positive values indicate dominant 4 Hz power, negative values dominant 2 Hz power. A paired *t*-test, together with one-sample tests against zero for each condition (see the Results), confirmed a significant bias toward 4 Hz in the Low-tone compared with the High-tone condition (*p* < 0.0001, Cohen’s d = 1.11), underscoring stream-specific entrainment.

To test whether oculomotor dynamics track the perceived auditory rhythm, we performed a spectral analysis of eye-velocity modulation. For each 3 s epoch we computed the power-spectral density (PSD), then averaged PSDs across participants within each perceptual condition. We focused on the 2 Hz and 4 Hz components that correspond to the repetition rates of the high- and low-tone streams (see *Methods*). Figure 3A displays the group-mean PSDs.

A two-way repeated-measures ANOVA with factors *Frequency* (2 Hz vs. 4 Hz) and *Condition* (Low-tone, High-tone, All) revealed a significant main effect of frequency, *F*(1, 84) = 23.3 *p* < 0.00001, and a significant Frequency × Condition interaction, *F*(2, 84) = 13.2, *p* < 0. 00001. Post-hoc comparisons showed that the Low-tone and All conditions exhibited stronger 4 Hz power than 2 Hz power (*p* < 0.0001, **** and, *p* < 0.001, ***, respectively) but no reliable difference was found in the High-tone condition. To visualize the dominance of each frequency, we subtracted 2 Hz power from 4 Hz power for every participant (ΔPSD = PSD_4Hz_ – PSD_2Hz_). Figure 3B shows these individual ΔPSD values. Consistent with the instructed percept, ΔPSD was positive in the Low-tone condition, indicating stronger 4 Hz entrainment and negative in the High-tone condition, indicating stronger 2 Hz entrainment. A paired *t*-test on the Low- vs. High-tone ΔPSD confirmed this dissociation, *p* < 0.0001 and Cohen’s d = 1.11. One-sample *t*-tests against zero further characterized the direction of the effect within each percept category: Low (N = 15, mean ΔPSD = 0.12), *t*(14) = 3.79, *p* < 0.005 and High (N = 15, mean ΔPSD = −0.032), *t*(14) = −0.81, *p* > 0.1. Together, these results show that fixational eye-movement (eye-velocity) dynamics synchronize with the dominant acoustic rhythm of the currently perceived stream.

Participants often reported difficulty sustaining a single auditory stream for 20 s trial, and ΔPSD in Figure 3B showed substantial inter-individual variability. Such variability could reflect intra-trial perceptual fluctuations (as seen in other bistable phenomena like in binocular rivalry), stable individual differences, or a combination of both. To directly investigate potential intra-trial dynamics, we examined how eye-movement power at 2 Hz and 4 Hz varied over time.

**Figure 4:**
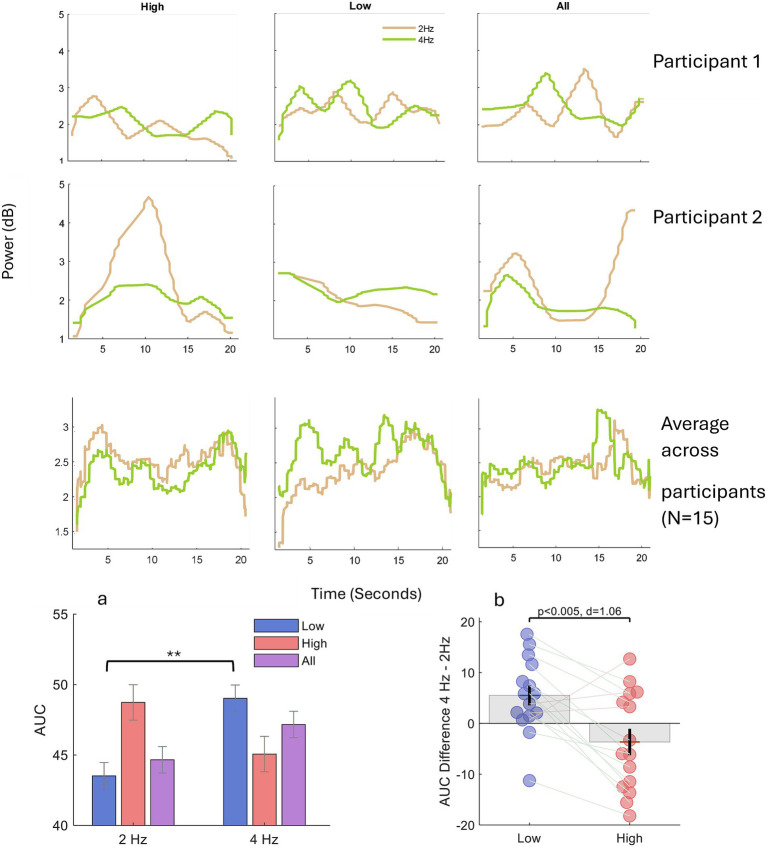
Experiment 1: temporal fluctuations of oculomotor entrainment. Upper panel Time-resolved power at 2 Hz (green) and 4 Hz (orange) over the 20 s trial, estimated with 3 s sliding window (mid-window time-points shown). Traces are shown for each perceptual condition (*High-tone*, *Low-tone*, *All*) together with two representative participants illustrating comparatively stable versus variable stream dominance. Group averages reveal persistent 2 Hz dominance when the High-tone stream is attended, persistent 4 Hz dominance when the Low-tone stream is attended and alternating (“rivalrous”) dominance when the full sound pattern is attended. Lower Panel. **(a)** Difference in area under the curve (AUC) between 4 Hz and 2 Hz power for each condition. A two-way repeated-measures ANOVA (Condition × Frequency) showed a significant interaction (F(2,84) = 9.74, *p* < 0.0005). Post-hoc tests indicated a larger 4 Hz AUC in the Low-tone condition (*p* < 0.01, **). **(b)** Individual AUC differences (4 Hz–2 Hz) for the Low- versus High-tone conditions. Twelve of fifteen participants exhibited greater 4 Hz than 2 Hz power when perceiving the Low-tone stream, yielding a significant paired-sample difference (*p* < 0.005, Cohen’s d = 1.06; see the Results for additional statistics).

The upper panel of Figure 4 plots the fluctuating power at 2 Hz and 4 Hz across the 20 s trials, averaged within participant and condition. Traces are shown for each condition (High-tone, Low-tone, All), along with two representative participants to highlight individual differences. Group averages reveal a dominant 2 Hz component during High-tone perception, a dominant 4 Hz component during Low-tone perception, and an alternating pattern when both streams are present in the All condition. The lower panel quantifies these trends by comparing the area under the curve (AUC) for 2 Hz versus 4 Hz power.

A two-way repeated-measures ANOVA yielded a significant Condition × Frequency interaction, *F*(2, 84) = *9.74*, *p* < 0.0005. Post-hoc tests confirmed a larger 4 Hz AUC in the Low-tone condition (*p* < 0.01, **). Consistent with this, a paired *t*-test on individual AUC differences (4 Hz – 2 Hz) showed that 12 of 15 participants exhibited stronger 4 Hz than 2 Hz power when attending to the Low-tone stream (*p* < 0.005, Cohen’s d = 1.06). These results underscore dynamic, stream-specific entrainment of oculomotor activity over the course of each trial. One-sample *t*-tests against zero further characterized the direction of the effect within each percept category: Low (N = 15, mean ΔAUC = 5.5), *t*(14) = 2.9, *p* < 0.05 and High (N = 15, mean ΔAUC = –3.6), *t*(14) = −1.46, *p* > 0.1.

### Experiment 2

**Figure 5:**
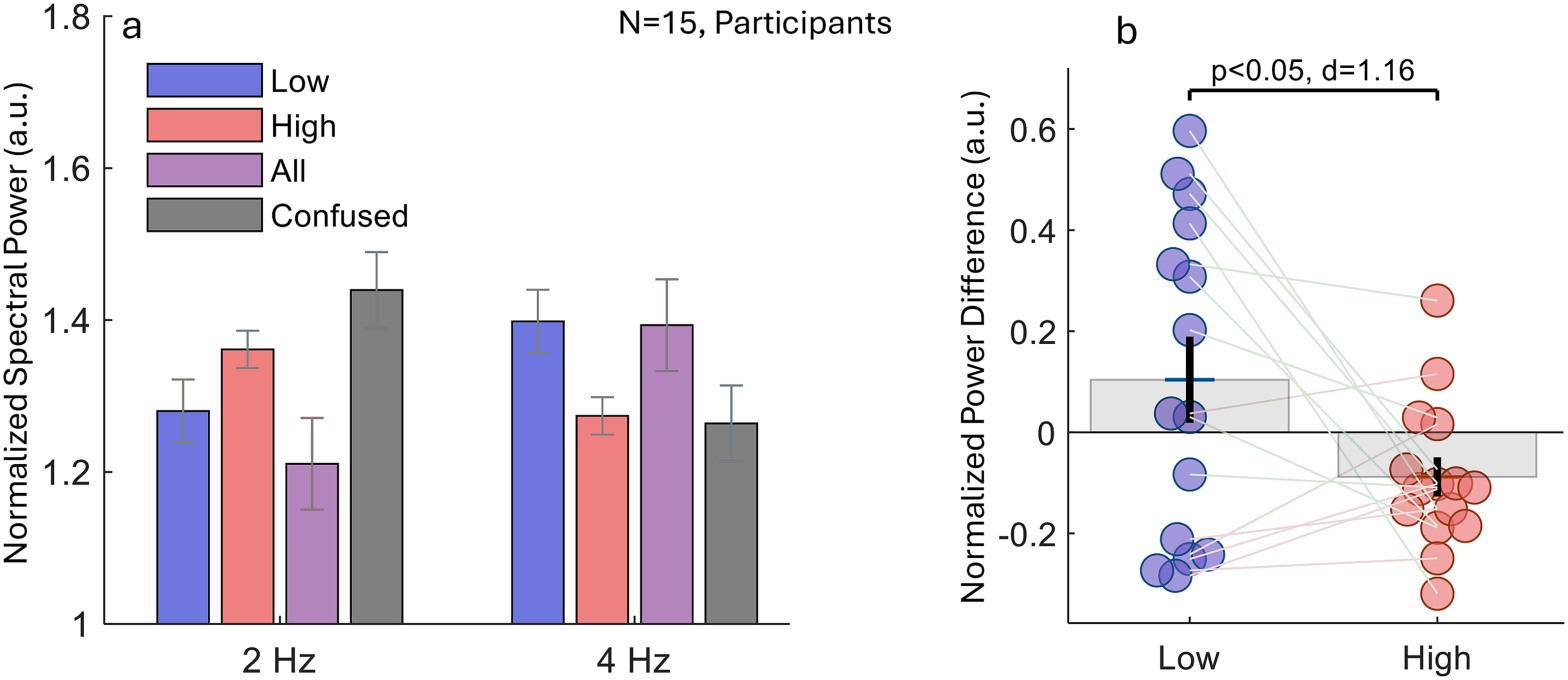
Experiment 2: spectral signatures of oculomotor entrainment during subjective stream reports. **(a)** Group-mean power-spectral density (PSD) of eye-velocity fluctuations, normalized within participants (see the Methods). When listeners reported hearing the *High-tone* stream, power was concentrated at 2 Hz, whereas reports of the *Low-tone* stream or the full pattern *All-tones*, showed enhanced 4 Hz power. A two-way repeated-measures ANOVA (Condition × Frequency) yielded a non-significant trend of condition (*F*(3,109) = 2.15, *p* < 0. 1) and a highly significant Condition × Frequency interaction (*F*(3,109) = 9.26, *p* < 0.0001). **(b)** Individual PSD differences (4 Hz–2 Hz) for the Low-tone versus High-tone percepts. Positive values indicate dominant 4 Hz power. A paired-sample *t*-test indicated a significant trend toward stronger 4 Hz than 2 Hz entrainment during Low-tone compared with High-tone perception (*p* < 0.05, Cohen’s d = 1.16; see the Results for additional statistics). Together, these findings replicate the stream-specific oculomotor entrainment observed in Experiment 1.

A second experiment used the same ABA- auditory pattern but changed the procedure: each 5 s stimulus block was immediately followed by a percept report (High-tone, Low-tone, All, or Confused when perception was unstable; see Methods). Across participants, the average number of trials per perceptual category (M ± SD) was: All (19.3 ± 18.0), High (47.2 ± 30.5), Low (42.0 ± 29.1), and Confused (17.3 ± 14.4), providing sufficient sampling for all conditions while highlighting greater variability for the Confused percept. For every 5 s epoch we computed the power-spectral density (PSD) of eye velocity and extracted the 2 Hz and 4 Hz components. Figure 5A shows the group means. A two-way repeated-measures ANOVA (*Condition* × *Frequency*) yielded a non-significant trend for the main effect of condition, *F*(3,109) = 2.15, *p* < 0. 1, alongside a robust Condition × Frequency interaction, *F*(3,109) = 9.26, *p* < 0.0001. Post-hoc comparisons indicated a reliable 4 Hz > 2 Hz bias only in the All condition (*p* < 0.05) whereas the Confused condition showed the opposite pattern (2 Hz > 4 Hz, *p* < 0.05). Figure 5B plots the individual frequency differences (ΔPSD = PSD_4Hz_–PSD_2Hz_). ΔPSD was positive for the Low-tone percept and negative for the High-tone percept, consistent with stronger oculomotor entrainment at 4 Hz versus 2 Hz when the Low-tone stream was dominant (*p* < 0.05, paired *t*-test). Finally, one-sample *t*-tests against zero confirmed the direction of the ΔPSD bias within each percept category: Low (N = 15, mean ΔPSD = 0.104), *t*(14) = 1.26, *p* = 0.228 and High (N = 15, mean ΔPSD = −0.088), *t*(14) = −2.35, *p* = 0.034.

## Discussion

Across two experiments, we asked whether fixational eye movements align with the perceived temporal structure of an ambiguous ABA–sequence under unchanging acoustics. When listeners perceived the high-tone stream, eye-movement velocity spectra tended to be weighted toward the 2-Hz component, whereas perception of the low stream and the integrated (“All”) percept was accompanied by enhanced 4-Hz power.

Importantly, the strength of this dissociation depended on procedure. In Experiment 1 (explicit attentional instructions), within-condition tests showed that the Low-tone and All conditions exhibited greater 4 Hz than 2 Hz power, whereas the High-tone condition showed no reliable 4–2 Hz difference. Complementing these within-condition effects, the between-condition summary metric ΔPSD = PSD_{4 Hz} − PSD_{2 Hz} provided a compact index of perceptual state: ΔPSD differed robustly between Low and High, being more positive for Low-tone than for High-tone perception. In Experiment 2 (subjective reports), this Low–High separation in ΔPSD was preserved. Separately, within-condition post-hoc comparisons in Experiment 2 clarified how the component frequencies behaved across reported states: a reliable 4 Hz > 2 Hz bias emerged most clearly for the All percept, whereas Confused epochs showed the opposite 2 Hz > 4 Hz pattern. Together, these findings show that involuntary fixation dynamics reweight the 2- and 4-Hz temporal components of an ambiguous auditory sequence in a percept-dependent manner, supporting fixation dynamics as a potential report-free tracer of the internal organization of sound.

Converging empirical observations support a percept-contingent account. First, although microsaccade (MS) probability showed rhythm-specific peaks, one every 0.5 s (2 Hz) or two every 0.5 s (4 Hz) depending on perceptual condition, these modulations did not reach significance and are presented only to illustrate MS timing (Figure 2A). Therefore, we asked whether the latency of the first MS following post-stimulus inhibition (msRT) would reveal a discrepancy between percept conditions. Indeed, msRT was significantly shorter when listeners tracked the low-frequency stream than when they tracked the high-frequency stream. This latency shift is consistent with percept-dependent modulation of oculomotor inhibition timing and may suggest that the oculomotor system paces microsaccades to sample the attended stream, releasing inhibition preferentially during moments of reduced informational value (Figure 2B), although the present data primarily establish the msRT difference itself. Across the block, first-MS latency also showed a modest within-block trend (significant in Low, marginal in High, absent in All; Figure 2c), suggesting that MS timing can drift over the course of stimulation, consistent with gradual adaptation or percept stabilization.

Second, in parallel with microsaccade timing, the normalized spectral power of eye-movement velocity, that is largely dependent on MS dynamics, showed clear entrainment to the perceived rhythm of the sequence. Power increased selectively at the percept-relevant frequency: 2 Hz when listeners perceived the high-tone stream, and 4 Hz when they perceived the low or integrated (“All”) stream. These effects emerged under directed-attention instructions (Experiment 1, Figure 3) and were replicated when percepts were freely reported (Experiment 2, Figure 5), where the 2–4 Hz redistribution tracked perceptual state across experiments and was well captured by the ΔPSD (4–2 Hz) index, which robustly separated Low from High percepts.

Experiment 1 revealed a significant interaction between frequency (2 vs. 4 Hz) and perceptual condition. In the Low-tone condition, power at 4 Hz was significantly greater than at 2 Hz. In contrast, the High-tone condition was noisier and the 2–4 Hz difference did not reach significance. One plausible reason is stimulus-driven multistability: the ABA– sequence can support multiple embedded organizations and listener-specific parsing strategies (Denham et al., 2014), such that attending to the High stream may bias dynamics toward 2 Hz while retaining higher-frequency “anchors,” reducing the sensitivity of a simple within-condition post-hoc contrast even as ΔPSD robustly captures the overall Low–High separation. This variability was also evident in the individual data: 6 of 15 participants showed relatively high 4 Hz power even in the High-tone condition (Figures. 3B, 4B). Notably, 5 of these 6 participants also showed higher 4 Hz power in the Low-tone condition than the rest of the group, suggesting stable individual differences, such as a higher baseline microsaccade rate or a different intrinsic pacing of microsaccades, rather than a condition-specific effect. Interestingly, the All-tone condition showed significantly greater 4 Hz than 2 Hz power. Based on our previous work, this pattern can emerge for longer stimuli (e.g., >300 ms), where the microsaccade rate exhibits a second phase of inhibition followed by a rebound induced by the stimulus offset (Kadosh and Bonneh, 2022b).

Because sustaining a single percept for 20 s is difficult, we examined how these rhythmic components evolved over time. A time-resolved spectral analysis using a 3 s sliding window revealed that the relative dominance of 2 Hz versus 4 Hz power waxed and waned across each trial, consistent with spontaneous perceptual alternations. Quantifying the area under the curve (AUC) for the 2 Hz and 4 Hz components confirmed that their difference replicated the main effect observed in the global spectral analysis. These dynamics align with the well-established temporal statistics of auditory bistability: after the initial build-up, dominance durations are often on the order of several seconds but can vary widely with stimulus regime, task demands, and stable individual differences, spanning from a few seconds to tens of seconds (Rankin et al., 2015; Deike et al., 2012, 2015; Denham et al., 2014; Sanders et al., 2018; Curtu et al., 2019). Importantly, because our estimates are based on a 3 s window and do not include continuous percept reports, we do not infer precise switching times from the eye-movement signal; rather, we view the observed waxing-and-waning as consistent with the possibility that fixation rhythms may track perceptual fluctuations on seconds-scale timescales.

Finally, grouping the short (5 s) trials in Experiment 2 by self-reported percept yielded the same overall pattern: the 4 Hz − 2 Hz power difference (ΔPSD) reversed sign with the dominant percept. The Low- and High-tone conditions showed effects in the expected directions, but only the High-tone condition reached significance in this experiment. The All-tone condition also showed a significant 4 Hz > 2 Hz bias, consistent with Experiment 1 (Figure 5A). The observed 4-Hz weighting in the All (“galloping”) percept is consistent with a within-cycle “two-anchor” parsing strategy within each 500-ms triplet, while recognizing that ABA– sequences can support multiple embedded organizations and listener-specific grouping strategies (Denham et al., 2014). Consistent with this, during piloting/debriefing several listeners described following the All percept by anchoring attention to the triplet onset and a later within-cycle landmark, which would naturally favor a 4-Hz subdivision.

Notably, epochs labeled “Confused” showed significantly greater 2 Hz than 4 Hz power.

Several factors could contribute to this pattern, most plausibly rapid alternations/weak dominance within the 5-s epoch and associated decision uncertainty, and in some cases brief attentional lapses. Accordingly, we interpret Confused reports not as a stable third percept but as transitional or unreliably labeled intervals, as commonly treated in prior streaming work (Rankin et al., 2015; Sanders et al., 2018; Curtu et al., 2019; Deike et al., 2012, 2015). In this regime, fixation dynamics may revert toward the typical microsaccade rhythm (approximately 1–3 per second).

### Percept-dependent oculomotor inhibition and rhythmic organization

Building on evidence that microsaccade dynamics index covert attention, temporal expectation, salience/deviance, and cognitive load/effort (Engbert and Kliegl, 2003; Yuval-Greenberg et al., 2014; Amit et al., 2019; Bonneh et al., 2013; Kadosh and Bonneh, 2022a, 2022b; Widmann et al., 2014; Contadini-Wright et al., 2023; Siegenthaler et al., 2014), we show that fixational eye movements are phase-locked to the perceived auditory rhythm, tracking the internal organization of sound rather than its external timing. Work on oculomotor inhibition demonstrates that auditory regularities automatically shape fixational behavior and index salience/deviance without overt reports (Kadosh and Bonneh, 2022a, 2022b), while temporal attention stabilizes fixation at expected moments, over and above predictability, revealing a top-down contribution to microsaccade suppression (Denison et al., 2019). Consistent with this framework, microsaccadic/oculomotor inhibition precedes predictable auditory targets, and rapid ocular “freezing” scales with bottom-up salience (Abeles et al., 2020; Zhao et al., 2019). Recent work further dissociates temporal expectations in oculomotor terms, with hazard-based expectation altering MS dynamics even without focused attention, elevating MS rate in the inhibitory window, delaying and reducing the rebound, and shifting the last pre-target MS, whereas precision-based expectation shows no main effect unless temporal attention is deployed, aligning MS modulations to the expected moment (Duyar and Carrasco, 2025). Beyond stimulus cadence and temporal expectations, oculomotor inhibition is also shaped by abstract linguistic organization, showing sensitivity to morphology-driven structure and lexical parsing, implicating higher-order lexical evaluation (Kadosh et al., 2025).

In our paradigm, where physical stimulation is constant, percept-contingent shifts in MS probability, msRT, and the spectral power of eye-movement velocity (dominated by MS) indicate that entrainment follows an internal temporal matrix aligned with the perceived stream (2 vs. 4 Hz), not stimulus cadence or generic salience.

### Active sensing and integration with temporal selection

Within an active-sensing framework, motor-derived predictions set the phase of sensory sampling and bias neural gain toward expected events; in audition, slow delta–beta coupling over sensorimotor cortex can feed predictive signals back to auditory cortices, enhancing processing at specific phases while listeners track regular structure, thus situating temporal attention within a motor–sensory loop (Lakatos et al., 2008; Schroeder and Lakatos, 2009; Morillon and Baillet, 2017; Lakatos et al., 2019; Coull et al., 2024, in *Neurobiology of Interval Timing*, 2nd ed.). Converging evidence indicates that this loop is instantiated within a core cortico–basal ganglia–thalamo–cortical timing network that supports both perceptual and motor timing, providing a plausible substrate for motor-to-sensory predictive control in the time range relevant to our task (Merchant and de Lafuente, 2024; see also Coull et al., 2024). In this loop, the observed alignment of MS probability, msRT, and spectral power with the perceived 2–4 Hz rhythm is precisely what one would expect if fixational dynamics are integrated with temporal selection, whereby the brain rhythmically coordinates perception and action (Lakatos et al., 2019). By timing microsaccades away from perceptually informative moments, the oculomotor system may implement a temporal sampling strategy that rhythmically gates sensory input in line with perceived structure, making fixational eye movements a rapid, involuntary readout of the brain’s internal temporal organization of auditory scenes; more broadly, MS participate in a closed perception–action loop in which motor commands shape sensory sampling and sensory outcomes update motor control (Ahissar and Assa, 2016; Gruber and Ahissar, 2020; Ahissar et al., 2016). Corollary-discharge signals from rhythmic motor activity can enhance auditory processing at specific phases, consistent with the present entrainment effects (Morillon et al., 2014; Morillon and Baillet, 2017; Coull et al., 2024).

### Perceptual punctuation: ISI as a motor–perceptual sampling unit

Although appropriately timed fixational eye movements are known to prevent fading and enhance spatial detail in vision (Rucci and Victor, 2015; Poletti and Rucci, 2016; Martinez-Conde et al., 2013; Intoy and Rucci, 2020), the present findings provide evidence that fixational eye-movement timing is coupled to the *perceived* temporal structure of an auditory stream under unchanged acoustics. In this sense, our results are consistent with the idea that the oculomotor system may resample in time relative to expected acoustic onsets. The inter-saccadic interval (ISI) may thus serve as a candidate internal timing unit within a Motor → Sensory → Motor loop, consistent with increased fixational stability when voluntary temporal attention targets expected moments and with pre-target microsaccadic/oculomotor inhibition before predictable auditory and tactile targets (Denison et al., 2019; Abeles et al., 2020; Badde et al., 2020), and with rhythmic accounts linking saccadic suppression to oscillatory modulations of sensitivity, attentional rhythms, and microsaccade–cortical coupling (Benedetto and Morrone, 2017; Hogendoorn, 2016; Bosman et al., 2009; Lowet et al., 2016; Lowet et al., 2018).

We therefore introduce the “perceptual punctuation” hypothesis as a post-hoc conceptual extension rather than a direct inference from the current data. Because each MS transiently suppresses visual sensitivity for tens of milliseconds (Hafed and Krauzlis, 2010; Hass and Horwitz, 2011; Greilich et al., 2024; Beeler, 1967), one possibility is that aligning MS to the perceived auditory rhythm could relegate these low-gain intervals to relatively lower-information phases, creating brief “rests” between perceptual “notes.” Importantly, we did not measure visual sensitivity in the present experiments, so this functional account remains speculative and requires targeted testing.

Similar principles govern other ocular events: eyeblinks are timed to narrative or speech pauses (Nakano et al., 2009; Nakano and Kitazawa, 2010), and small oculomotor events can bias perceptual switches in visual bistability (van Dam and van Ee, 2005; Bonneh et al., 2010; Otero-Millán et al., 2012). Finally, links between large saccades and subjective time (saccadic chronostasis) underscore the broader connection between oculomotor programming and temporal perception, in line with an embodied, motor-anchored account of timing (Yarrow et al., 2004; Merchant and Yarrow, 2016; Merchant and de Lafuente, 2024; Kresevic et al., 2016; Morrone et al., 2005; Yarrow et al., 2001; Zhai et al., 2024).

To illustrate the intuition, consider a galloping horse: if microsaccade timing were locked to the gallop, microsaccades would tend to fall between expected hoof-fall onsets, potentially preserving visual sensitivity when motion cues are most informative. The same logic could extend to other salient or behaviorally relevant sources (e.g., the March rhythm of an approaching animal), where an auditory beat might entrain a visual sampling schedule. However, these examples are illustrative, and the present study does not establish cross-modal consequences of such alignment.

Mechanistically, the known sensory costs of each MS, tens of milliseconds of reduced visual sensitivity, provide a plausible rationale for why such alignment to the perceived auditory object/stream (Bregman, 1990) could create brief ‘rests’ between perceptual notes.

In this view, perception unfolds like a sentence with its own prosody: blinks can act as full stops that briefly halt sampling; microsaccades behave like commas that segment the flow without interrupting it, spacing sensory uptake into meaningful units. Critically, this “punctuation” framing yields concrete, falsifiable predictions and offers a compact grammar for how the brain times what it sees to what it hears. A decisive test is whether entraining MS to an auditory stream shifts simultaneous visual sensitivity, revealing cross-modal trade-offs. A complementary prediction is that experimentally manipulating the auditory meter/predictability, together with the rhythmic characteristics of the visual scene, will reshape the timing of these putative punctuation marks.

This view accords with converging evidence that low-frequency neural entrainment provides a mechanism for temporal selection and multisensory binding (Lakatos et al., 2008; Schroeder and Lakatos, 2009; Mercier et al., 2015; Noesselt et al., 2007), linking the alignment of neural phase to the integration of sensory streams (Denison et al., 2013; Fujisaki and Nishida, 2005, 2007). Complementary oculomotor studies show that auditory deviance and salience modulate oculomotor inhibition (Kadosh and Bonneh, 2022a, 2022b; Widmann et al., 2014), while listening effort alters microsaccade dynamics independently of arousal (Contadini-Wright et al., 2023).

Together, these results motivate the possibility that fixational eye movements participate in a multimodal sampling schedule governed by low-frequency temporal prediction, a hypothesis that the present findings support at the level of timing alignment, but whose functional consequences remain to be demonstrated.

### Limitations and future directions

This study has several limitations. The sample size was modest, and only a single bistable sequence was tested; future work should use larger cohorts and more naturalistic acoustic scenes to assess generality. Our analyses were block-based, but state-resolved approaches centered on spontaneous perceptual switches could reveal how oculomotor timing evolves during dominance transitions. Combining EEG with eye-tracking may determine whether MS-locked cortical oscillations anticipate perceptual shifts and whether known modulators of auditory bistability influence the ocular rhythm. Finally, causal manipulations are needed to test whether MS simply read out perceptual organization or help shape it, for example, by entraining MS to an auditory beat while measuring phase-dependent changes in visual sensitivity.

## Data Availability

The raw data supporting the conclusions of this article will be made available by the authors, without undue reservation.
